# Supplementary material for: Modulus adaptive lubricating prototype inspired by instant muscle hardening mechanism of catfish skin
Source: Nat Commun. 2022 Jan 19;13:377. doi: 10.1038/s41467-022-28038-9 (PMC8770490; doi:10.1038/s41467-022-28038-9)
Supplement: Supplementary file 1 — Supplementary Information [file 41467_2022_28038_MOESM1_ESM.pdf]

Modulus adaptive lubricating prototype inspired by instant muscle hardening mechanism of catfish skin

Yunlei Zhang[1,2], Weiyi Zhao[1,2], Shuanhong Ma[1]*, Hui Liu[1,2], Xingwei Wang[1], Xiaoduo Zhao[1], Bo Yu[1], Meirong Cai[1], Feng Zhou[1]*

[1]State Key Laboratory of Solid Lubrication, Lanzhou Institute of Chemical Physics, Chinese Academy of Sciences, Lanzhou, 730000, China

[2]College of Materials Science and Opto-Electronics Technology, University of Chinese Academy of Sciences, Beijing, 100049, China

*Correspondence authors email:

mashuanhong@licp.cas.cn (S.M.);zhouf@licp.cas.cn (F.Z.)

Supplementary Methods

1. Chemicals and materials:

Acrylic acid (AA, >99%, TCI), acrylamide (AAm, >99%, J&K Chemical Ltd.), N, N'-methylene diacrylamide (MBAA, >99%, Sinopharm Chemical Reagent Co. Ltd., China), Calcium acetate ($C_4H_6CaO_4$, >99%, Tianjin Chemical Reagents Corp.), Polydimethylsiloxane (PDMS) and curing agents (SYLGARD 184 kit, Dow corning, Midland, MI, USA), benzophenone (>99%, MACKLIN), α-Ketoglutaric acid (99%, J&K Chemical Ltd.), 2-Hydroxyethyl methacrylate (HEMA, 99%, J&K Chemical Ltd. 99%), 3-sulfopropyl methacrylate potassium (SPMA, >99%, TCI), sodium carbonate (Na_2CO_3, >99%, Sinopharm, China.), sodium chloride (NaCl, >99%, Sinopharm, China.) Dichloromethane and anhydrous methanol were purchased from Tianjin Chemical Reagents Corp. Dichloromethane was dried over CaH_2 before use. 2,2′-bipyridine (Bipy, 99%), copper (I) bromide (CuBr, 99%) were purchased from TCI Co., Ltd. CuBr was purified by stirring overnight in acetic acid. Plasticine was bought commercially (GB6675-2014).

2. Construction of artificial arm test platform and demonstration experiment. Programmable steering gear and circuit board was commercially purchased. The structure of artificial arm was produced by 3D printing. Contact pair of arm were made from PDMS. Data acquisition system was constructed by DAQ (NI9215 and NI cDAQ-9171, National Instrument). Force sensing device was was commercial purchased from Simbatouch, China. EZ-Test, SHIMADZU, was used as a stepper motor in experiment. A certain grasping force (400mN per arm) was obtained by

controlling the input signal of the artificial arm. The testing velocity was kept at 50 mm/min. The lubrication sample was pasted on both sides of the glass sheet for experiment at 20 °C and 80 °C. Toy submarine was commercially purchased. The driving force of toy submarine was 16mN. The lubrication sample was pasted on the submarine for experiment at 20 °C and 80 °C. A certain grasping force (200mN per arm) was obtained by controlling the input signal of the artificial arm. Toy tanks (40 g) was commercially purchased. The tilting Angle of the tilting platform is 45 °. The sample was attached to a sheet of glass. Andthe experimental temperature of the demonstration experiment is 20 °C and 80 °C.

3. Hertz contact analysis and Finite Element Analysis.

The contact stress and deformation analysis was based on Hertz contact theory.[1,2] Paraments in Hertz contact model were used from the experimental results in this work. Mechanical property paraments of hydrogels was obtained from related data in mechanical test and nano-indentation test. The modulus of MALH substrate (P(AAc-CaAc-co-HEMA-Br) hydrogel) at soft and rigid state was 0.3 MPa and 120 MPa, respectively. The surface modulus of lubricating layer of MALH at soft and rigid state was 0.06 MPa and 0.07 MPa, respectively. However, due to the substrates effect and mechanical trapping effect of MALH, the overall modulus of the lubrication layer was gradient. The modulus of this layer should be much higher when close to the substrates. Due to the limitation of experimental conditions, exact modulus gradient of lubricating layer was unknow. So, before a Finite Element Analysis, a simple analysis in Hertz contact model was used to identify the effect of

the lubricating layer in the whole MALH contact properties. And these experiment data and analysis data were used to help the Finite Element Analysis predicting the deformation, contact stress, contact radius more accurately. Finite Element Analysis was performed by Ansys 2020R1.

First, in order to simplify the analysis, we carried out a macrosopcis indentation test (indentation radius (*R*): 3 mm, load (*F*):1 N) and analyses MALH as a whole. Displacement (*d*) of Blank-S (Soft) in macroscopic sphere indentation was ~901 μm, and that of MALH-S was ~1017 μm. Displacement of Blank-R (Rigid) in macroscopic sphere indentation was ~23 μm, and that of MALH-R was ~55 μm. It was found that the displacement of MALH in soft state was ~100 μm deeper than that of blank in soft state after grafting polymer brush, while the displacement of MALH in rigid state was only ~22 μm more than that of blank in rigid state. This difference resulted from the substrates effect and mechanical trapping effect. The stiffening of the substrate resulted in an increase in the modulus gradient of the lubricating layer. This displacement data can be transformed into contact radius (a) by geometry condition in Hertz model.

$$a^2 = Rd. \qquad (1)$$

The effective modulus (E_{eff}) of the whole MALH can be calculated by,

$$E_{eff} = \frac{3FR}{4a^3}. \qquad (2)$$

And the contact stress distribution (*P*) was calculated by,

$$P_0 = \left(\frac{6FE_{eff}^2}{\pi^3 R^2}\right)^{\frac{1}{3}}. \qquad (3)$$

$$P = P_0(1 - \frac{r^2}{a^2})^{\frac{1}{2}}. \ (0 < r < a) \qquad (4)$$

In this simple analysis, the max contact stress (P_0) of MALH in soft and rigid state was ~0.106 MPa and ~2.885 MPa, respectively. However, this simple analysis took advantage of the effective modulus calculation of the whole MALH, ignoring the gradient of the lubrication layer. In the following FEA analysis, based on the displacement data and effective modulus data, we thought that the modulus of the lubricating layer in MALH-S increased slowly and became the same as the substrate after compression about 100%. For MALH-R, we assumed that the modulus increased dramatically and became the same as the substrate after compression about 30%. So, the paraments of surface lubricating layer were modified by the data in nanoindentation, the macrospic indentation test in Hertz analysis and the modulus of substrates in mechanical test and fitted by odgen model in ANSYS 2021R1. The substrate modulus data was the same as the mechanical test data. The load paraments was the same as the macroscopic indentation test in simulation. The Finite Element Analysis predicted that the contact stress of MALH at soft state and rigid state was was ~0.087 MPa and ~2.983 MPa, respectively. And in deformation results, soft MALH was also much larger than rigid MALH.

Supplementary Figures (Supplementary Figure 1-Supplementary Figure 14)

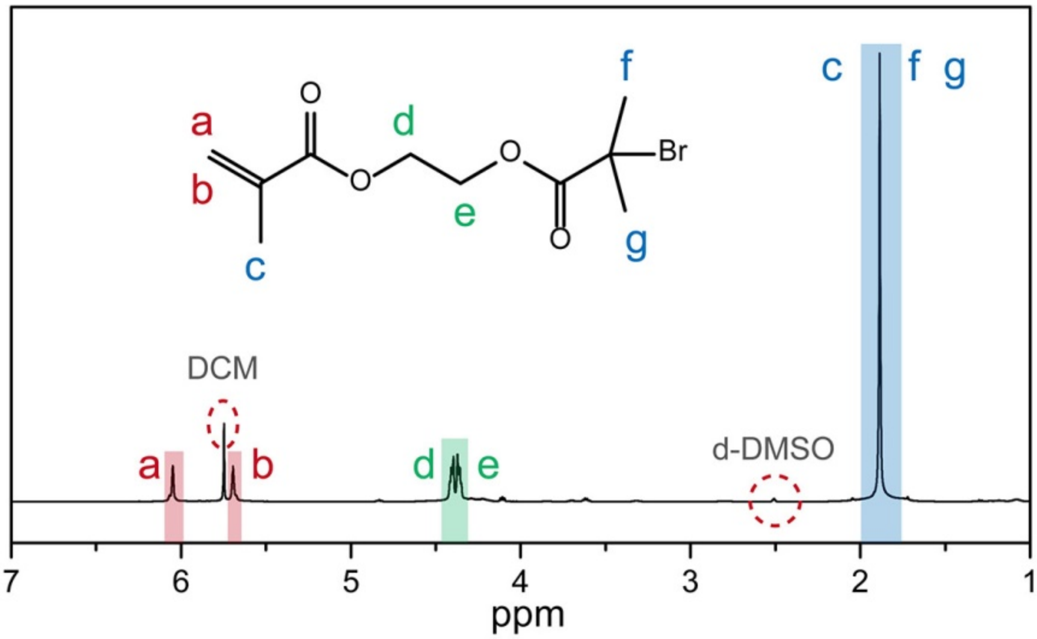

Supplementary Figure 1. Characterization of molecular structure of SSI-ATRP initiator by ^{1}H-NMR. The ^{1}H-NMR spectrum of SSI-ATRP initiator in d-DMSO solvent and the characteristic NMR peaks corresponding to SSI-ATRP initiator are clearly labeled.

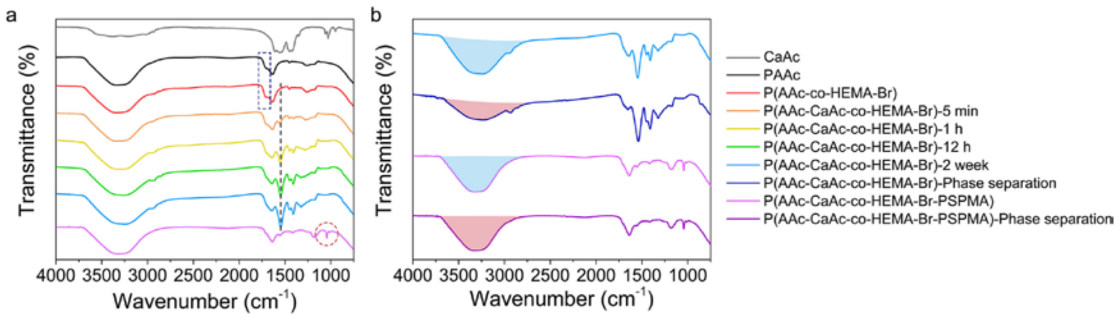

Supplementary Figure 2. ATR-FTIR spectra of different samples before and after phase separation. (a) ATR-FTIR spectra of CaAc, PAAc hydrogel, P(AAc-co-HEMA-Br) hydrogel, P(AAc-CaAc-co-HEMA-Br) hydrogel immersed in CaAc solution with different time, P(AAc-CaAc-co-HEMA-Br-PSPMA) hydrogel (MALH). (b) ATR-FTIR spectrum of P(AAc-CaAc-co-HEMA-Br) hydrogel and P(AAc-CaAc-co-HEMA-Br-PSPMA) hydrogel (MALH) before and after phase separation.

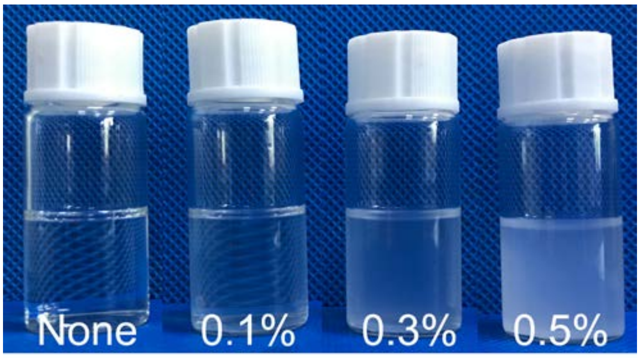

Supplementary Figure 3. States of monomer solution for preparing MALH samples. Photographs of the phase separation of the monomer solution with the increase of SSI-ATRP initiator content.

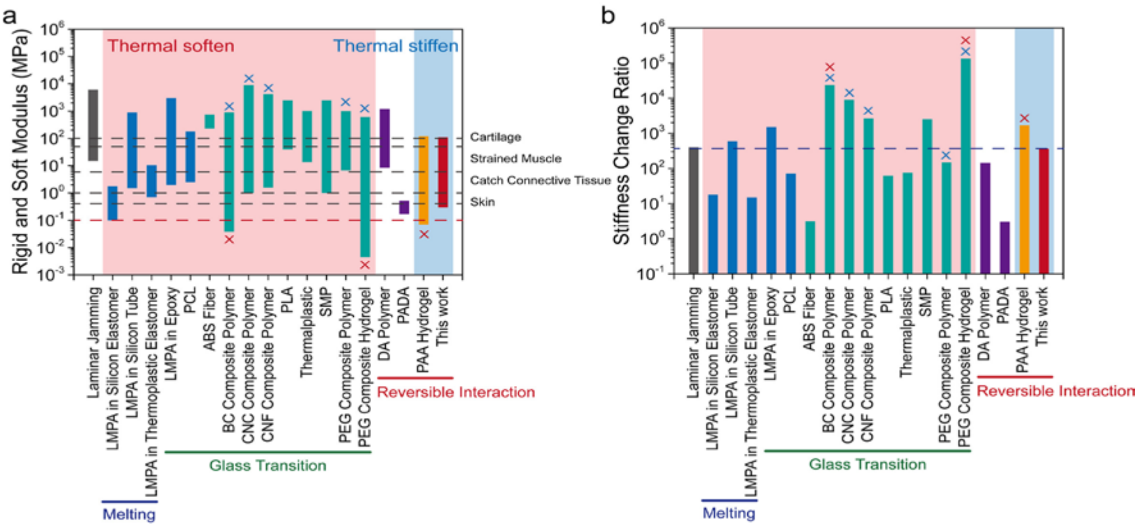

Supplementary Figure 4. Comparison of stiffness-change performance to representative results in literatures. (a) Comparison of modulus change range to the materials in the literature. (b) Comparison of stiffness change ratio to the materials in the literature. The variable stiffness materials in this figure, in order, include jamming techniques: layer jamming;[3] melting techniques: Low melting point alloy (LMPA) in silicon elastomer,[4] LMPA in silicone tubing,[5] LMPA in silicone thermoplastic elastomer,[6] LMPA in epoxy,[7] polycaprolactone (PCL) polymer,[8] glass transition techniques: Acrylonitrile butadiene styrene (ABS) polymer,[9] BC,[10] CNC,[11] CNF composite polymer,[12] polylactic acid (PLA) polymer,[13] thermoplastic,[14] shape memory polymer (SMP), [15] PEG composite polymer,[16] PEG composite hydrogel,[17] DA polymer,[18] PADA,[19] PAA hydrogel,[20] and this work: MALH. The red dotted line represents the appropriate load bearing capacity; The black dotted lines represent the modulus range of the tissue in the living organisms; The blue dotted line represents the change ratio of this work; the red cross indicates that the soft state is too weak to hold the load; the blue cross indicates that the material is not resistant to water.

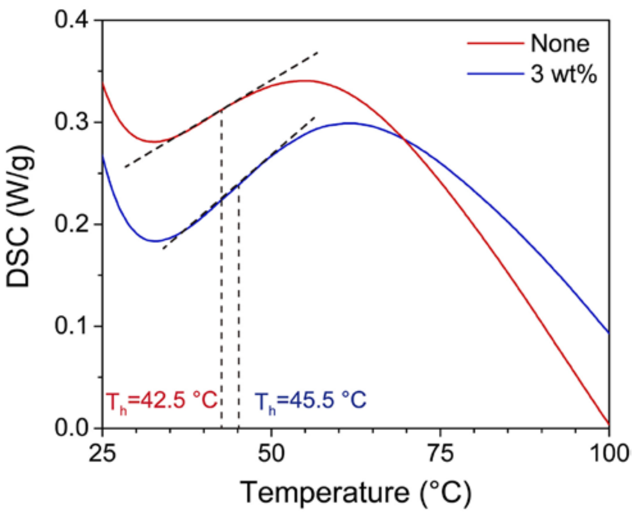

Supplementary Figure 5. Investigation of temperature transition point of phase separation. DSC curves of P(AAc-CaAc-co-HEMA-Br) hydrogel (red) and MALH-0.3wt% sample (blue). The coincidence points at maximum tangent lines in these curves represent the temperature of phase transition (T_h).

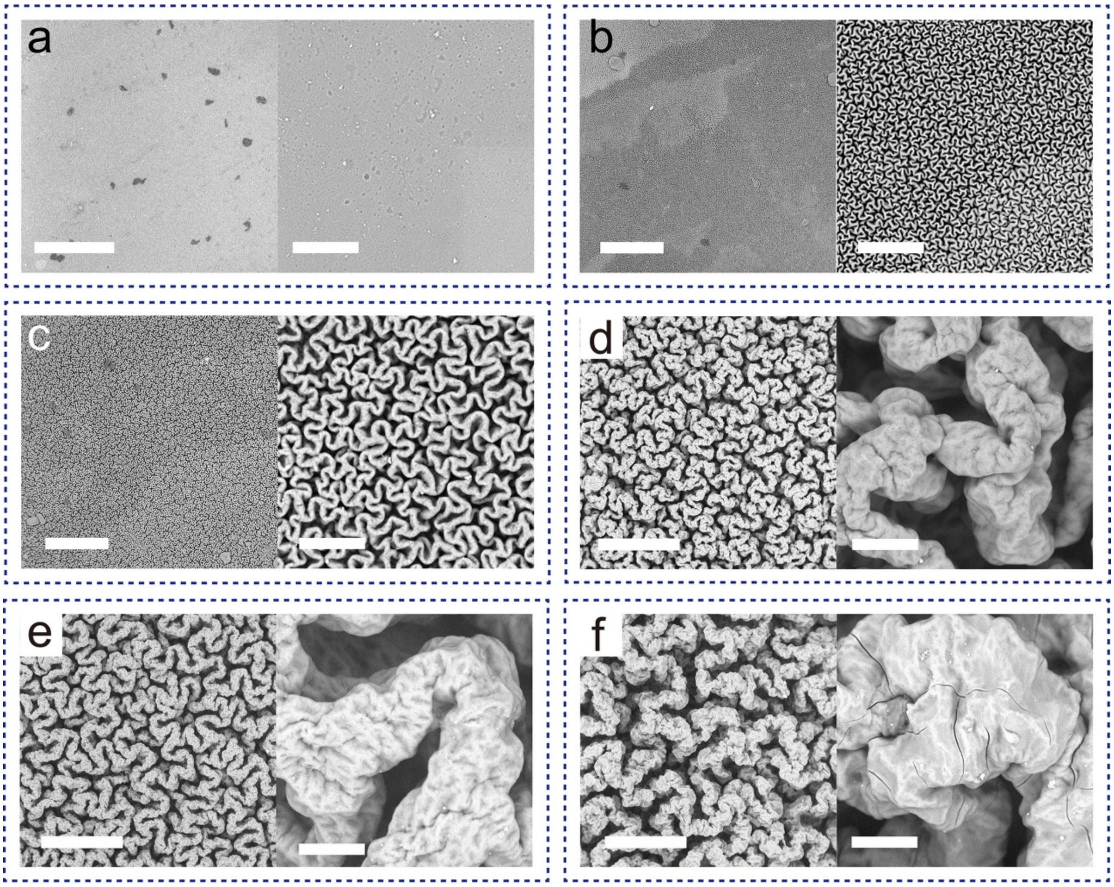

Supplementary Figure 6. Surface morphologies of MALH samples with different SSI-ATRP reaction time. (a) SEM images of composite hydrogel without polymer brush, left scale bar: 300 μm, right scale bar: 30μm. (b) SEM images of composite hydrogel with polymer brush, SSI-ATRP: 10 min, left scale bar: 200 μm, right scale bar: 30μm; (c) SSI-ATRP: 20 min, left scale bar: 300 μm, right scale bar: 30μm; (d) SSI-ATRP: 30 min, left scale bar: 300 μm, right scale bar: 30μm; (e) SSI-ATRP: 40 min, left scale bar: 300 μm, right scale bar: 30μm; (f) SSI-ATRP: 50 min, left scale bar: 300 μm, right scale bar: 30μm.

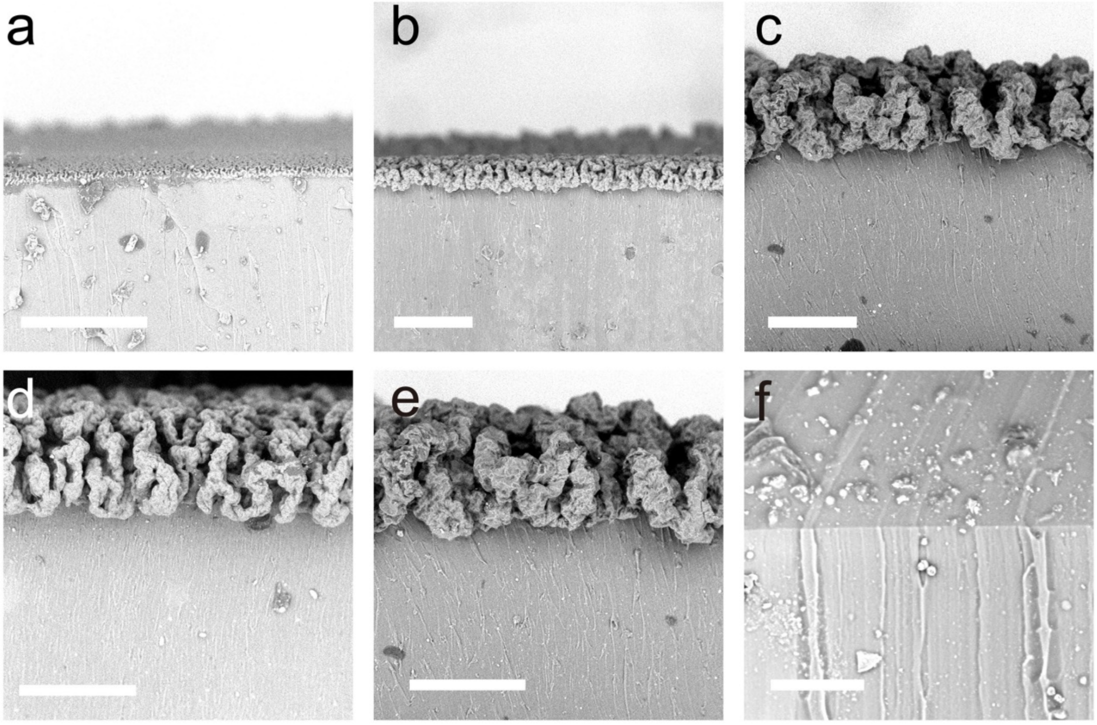

Supplementary Figure 7. Cross-sectional morphologies of MALH samples with different SSI-ATRP reaction time. (a) Cross-sectional of composite hydrogel with polymer brush, SSI-ATRP: 10 min, scale bar: 200 μm. (b) Cross-sectional of composite hydrogel with polymer brush, SSI-ATRP: 20 min, scale bar: 300 μm; (c) SSI-ATRP: 30 min, scale bar: 300 μm; (d) SSI-ATRP: 40 min, scale bar: 300 μm; (e) SSI-ATRP: 50 min, scale bar: 300 μm. (f) Cross-sectional of interface between PDMS-50:1 and PDMS-20:1, scale bar: 30 μm.

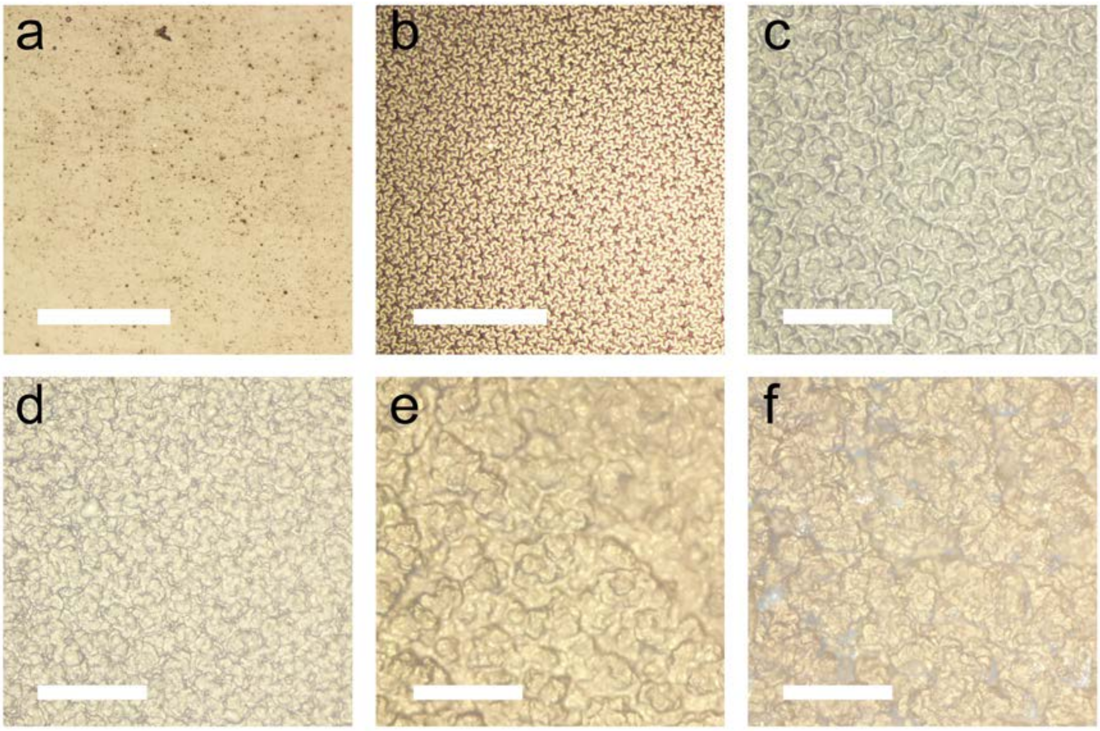

Supplementary Figure 8. Surface wet morphologies of MALH samples with different SSI-ATRP reaction time. (a) Optical image of composite hydrogel without polymer brush, scale bar: 200 μm. (b) Optical image of composite hydrogel with polymer brush, SSI-ATRP: 10 min, scale bar: 200 μm; (c) SSI-ATRP: 20 min, scale bar: 400 μm; (d) SSI-ATRP: 30 min, scale bar: 400 μm; (e) SSI-ATRP: 40 min, scale bar: 400 μm; (f) SSI-ATRP: 50 min, scale bar: 400 μm.

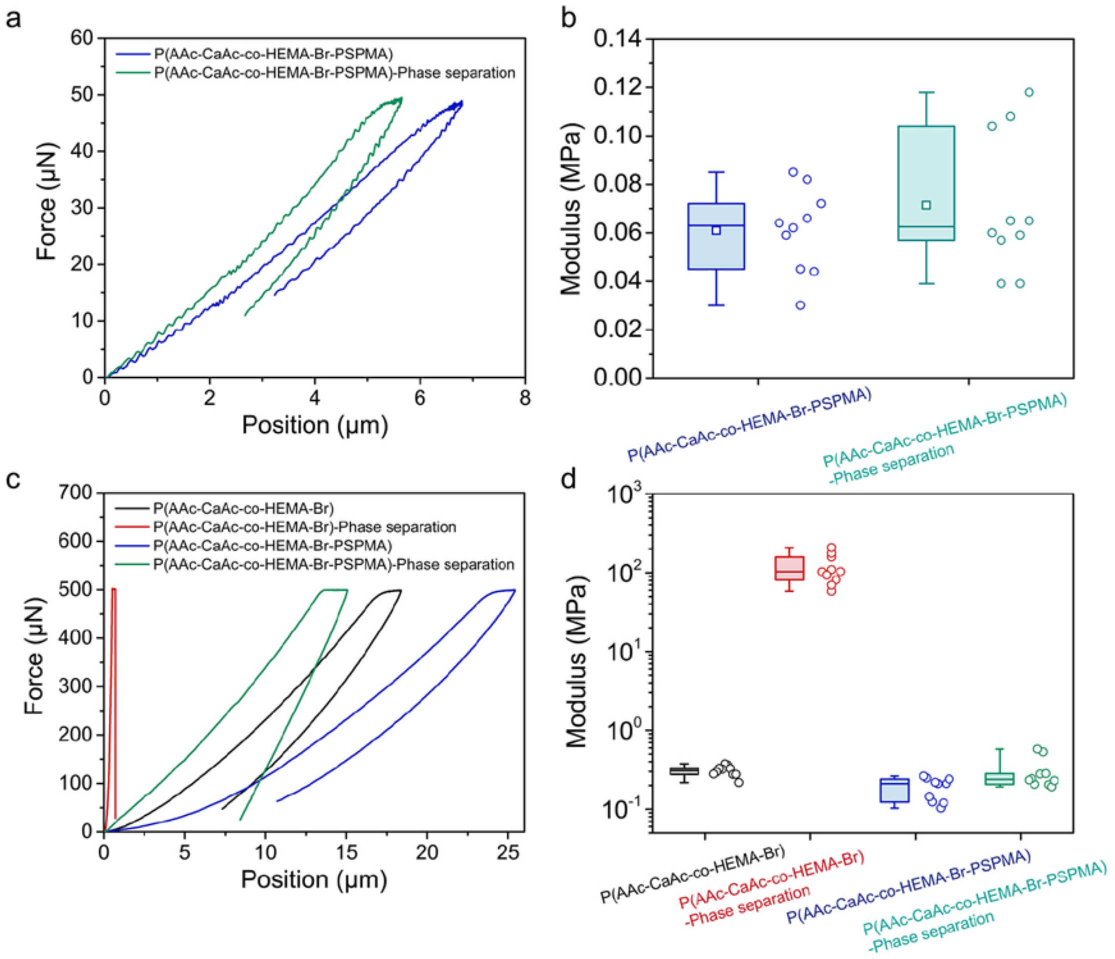

Supplementary Figure 9. Nano-indentation tests for MALH sample before and after phase separation (a, b) Force-position curves of nano-indentation tests for MALH sample before and after phase separation, and corresponding moduli results (load =50 μN), sample size n=10. (c, d) Force-position curves of nano-indentation tests for control sample without grafting polymer brush and MALH sample before and after phase separation, and corresponding moduli results (load =500 μN), sample size n=10.

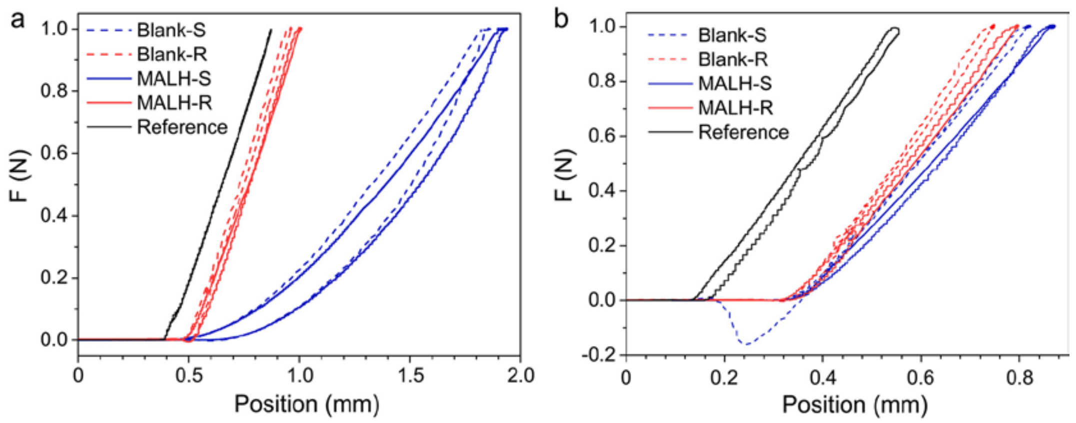

Supplementary Figure 10. Macro-indentation tests of samples at soft state (S-before phase separation) and rigid state (R-after phase separation). Force-position curves of blank sample without lubrication layer and MALH sample were obtained by using (a) sphere indentor and (b) flat plane indentor. The reference curve was the sensor displacement under testing load. The reference displacement of sphere indentation and flat plane indentation was 488.8±5 μm and 439.8 ±13 μm, respectively. Displacement of Blank-S in sphere indentation was ~901 μm, and that of MALH-S was ~1017 μm. Displacement of Blank-R in sphere indentation was ~23 μm, and that of MALH-R was ~55 μm. There data was fitted by Hertz model.

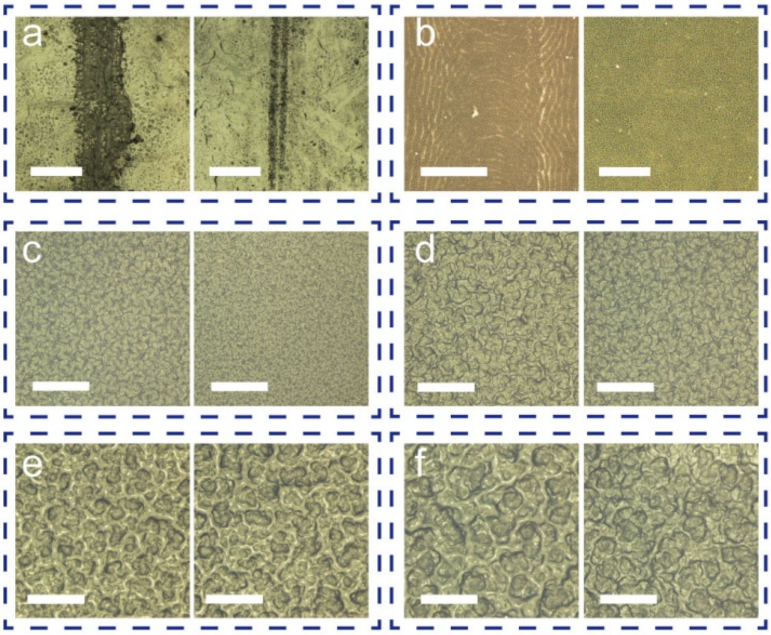

Supplementary Figure 11. Optical images of the surface morphologies of MALH samples with different SSI-ATRP reaction time after encountering friction test. (a) optical images of composite hydrogel without polymer brush after 10min friction test, left: friction test at 20 °C, scale bar: 400 μm, right: friction test at 80 °C, scale bar: 400μm. (b) optical images of composite hydrogel with polymer brush after 10min friction test, SSI-ATRP: 10 min, left: friction test at 20 °C, scale bar: 200 μm, right: friction test at 80 °C, scale bar: 200μm; (c) SSI-ATRP: 20 min, left: friction test at 20 °C, scale bar: 400 μm, right: friction test at 80 °C, scale bar: 400μm. (d) SSI-ATRP: 30 min, left: friction test at 20 °C, scale bar: 400 μm, right: friction test at 80 °C, scale bar: 400μm. (e) SSI-ATRP: 40 min, left: friction test at 20 °C, scale bar: 400 μm, right: friction test at 80 °C, scale bar: 400μm. (f) SSI-ATRP: 50 min, left: friction test at 20 °C, scale bar: 400 μm, right: friction test at 80 °C, scale bar: 400μm.

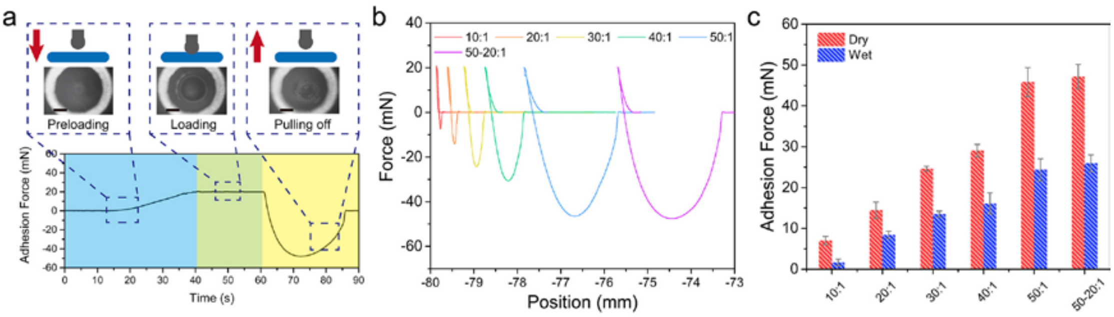

Supplementary Figure 12. Adhesion measurements of MASLD in dry and wet states. (a) Schematic diagram of normal adhesion force test process including three key stages: preloading, loading equilibrium and pulling off, along with corresponding adhesion force-time testing curves for sample (50-20:1), scale bar in optical images: 1mm. (b) The dry adhesion curve of PDMS with different proportion of cross-linking agent. (c) The dry, wet adhesion force of PDMS with different proportion of cross-linking agent, the data are presented as mean ± SD and sample size n=3.

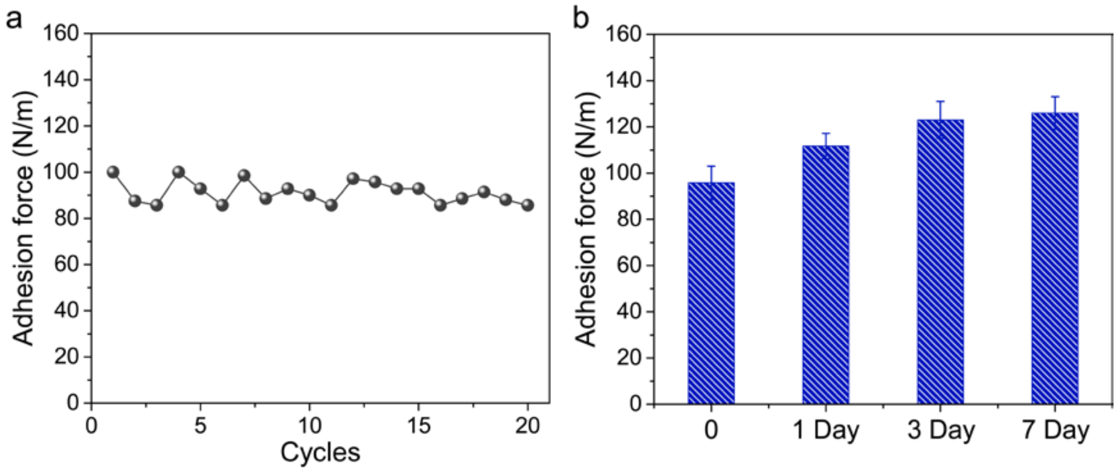

Supplementary Figure 13. Evaluation of adhesion stability of MASLD. Reversibility (a) and water resistance ability (b) of MASLD adhesion performance of MASLD, the data are presented as mean ± SD and sample size n=3.

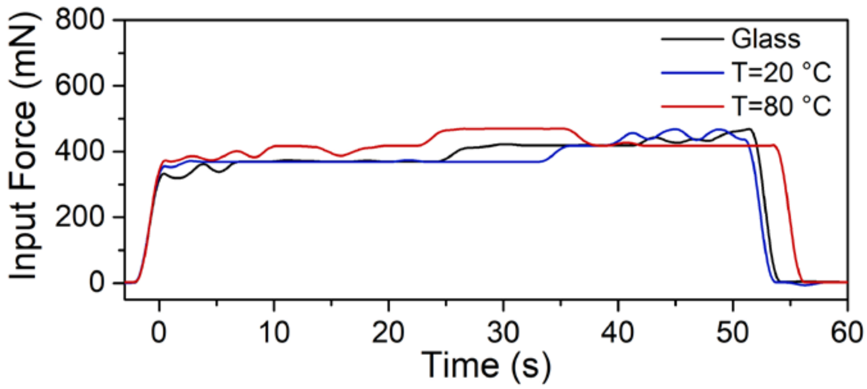

Supplementary Figure 14. The input force signals of artificial arms. The applying constant loading force cuves of per arm (about 400 mN) in force testing.

Supplementary Discussion (Supplementary Figure 4)

Although variable mechanically adaptive materials with switchable stiffness that enable dynamic load-bearing and impact-protective capability in response to external stimuli have been achieved through a variety of methods in soft-matter machines, biomedical engineering and wearable devices, such as 2-ureido-4-pyrimidone (UPy) based polymer, dynamic boron-oxygen bonds based shear stiffening gel (SSG), shear thickening fluid (STF), most of the materials mentioned above have disadvantages in intelligent lubrication systems for soft matter.[21-23] For example, the inevitable additional complex components in electro-rheological/magneto-rheological fluids system have severely limited their versatile applications.[24] Shear thickening fluid/gel systems require enormous external shear forces to stiffen, which is in contradiction to the small shear force in lubricating state. Some vacuum induced shape holding materials with switchable modulus such as laminar jamming also need complex equipment. [3,25] By contrast, MALH system is simple.

Solvent, humidity control strategies are not suitable for water lubrication system.[26] Among many solid–liquid phase change materials, low melting point alloys (LMPA) are particularly promising because of their significant modulus change and ideal melting temperature. However, the use of LMPA-based composites for actual applications can lead to difficulties due to the large surface tension of LMPA and weak interaction with polymer substrates.[4-7,17] And in Supplementary Figure 4, the modulus change range of these materials can't match the modulus of the biological tissue (0.1 MPa~100 MPa).[27] Glass transition polymers that become soft when heating them are considered as the most attractive options for building mechanically adaptive materials because of their large stiffness change. Although they have a wide range of variations, many of the materials cannot be used in an aquatic environment, have poor load-bearing capacity in soft state, or require complex molecular design.[9-17] In addition, the modulus change range of these materials also can't match the modulus of the biological tissue.[27] MALH can match the biological tissue modulus well, have a large change range and a good load bearing capacity.

Dynamic covalent polymer networks such as reversible Diels–Alder reaction can also achieve dynamic modulus changes, but also require complex molecular design.[18,19] Modulus change range of material (PAA hydrogel) based on cooperative effects of hydrophobic interaction and ionic interaction can match the modulus range of biological soft tissue.[20] However, PAA materials are not wear-resistant. MALH system is simple and has good lubrication performance.

Furthermore, most of the mechanically adaptive systems based on temperature control only stiffen in the absence of these stimulus, while soften in the presence of a stimulus. The systems in Supplementary Figure 4, red area, achieve a dramatic stiffness change thanks to these physical phenomena: the crystallization-melting transition and glass transition. This leads to most smart polymer materials that tend to soften rather than harden when thermal stimuli are applied. This is totally opposite of the stimulus input stiffening mechanism in fish skin or other stress reaction in biological system. Thermal stiffening mechanism of MALH can adapt to this bio-mechanism. Furthermore, most of soft robots are usually in soft state at room temperature and stiffen when needed. Therefore, MALH exhibited extraordinary potential application in soft robots.

Supplementary References

1. K. L. Johnson *Contact mechanics*. (Cambridge university press, 1987).
2. V. L. Popov *Contact mechanics and friction.* (Springer, 2010)
3. Y. S. Narang, J. J. Vlassak & R. D. Howe Mechanically versatile soft machines through laminar jamming. *Adv. Funct. Mater.* **28,** 1707136 (2018).
4. I. M. Van Meerbeek, et al. Morphing metal and elastomer bicontinuous foams for reversible stiffness, shape memory, and self‐healing soft machines. *Adv. Mater.* **28,** 2801-2806 (2016).
5. A. Tonazzini, et al. Variable stiffness fiber with self‐healing capability. *Adv. Mater.* **28,** 10142-10148 (2016).
6. S. Rich, S. H. Jang, Y. L. Park & C. Majidi Liquid metal‐conductive thermoplastic elastomer integration for low‐voltage stiffness tuning. *Adv. Mater. Technol.* **2,** 1700179 (2017).
7. T. L. Buckner, M. C. Yuen, S. Y. Kim & R. Kramer‐Bottiglio Enhanced Variable Stiffness and Variable Stretchability Enabled by Phase‐Changing Particulate Additives. *Adv. Funct. Mater.* **29,** 1903368 (2019).
8. M. A. McEvoy & N. Correll Thermoplastic variable stiffness composites with embedded, networked sensing, actuation, and control. *J. Compos. Mater.* **49,** 1799-1808 (2015).
9. M. C. Yuen, R. A. Bilodeau & R. K. Kramer Active variable stiffness fibers for multifunctional robotic fabrics. *IEEE Robotics and Automation Letters* **1,** 708-715 (2016).
10. Y. Qiu, et al. Dual-Stimuli-Responsive Polymer Composite with Ultrawide Tunable Stiffness Range Triggered by Water and Temperature. *ACS Appl. Polym. Mater.* **2,** 2008-2015 (2020).
11. M. Jorfi, M. N. Roberts, E. J. Foster&C. Weder Physiologically responsive, mechanically adaptive bio-nanocomposites for biomedical applications. *ACS Appl. Mater. Interfaces* **5,** 1517-1526 (2013).
12. J. R. Capadona, K. Shanmuganathan, D. J. Tyler, S. J. Rowan & C. Weder Stimuli-responsive polymer nanocomposites inspired by the sea cucumber dermis. *Science* **319,** 1370-1374 (2008).
13. Kamthai S, Magaraphan R. Thermal and mechanical properties of polylactic acid (PLA) and bagasse carboxymethyl cellulose (CMCB) composite by adding isosorbide diesters. *AIP conference proceedings. AIP Publishing LLC,* , **1664,** 060006 (2015)
14. A. Balasubramanian, M. Standish & C. J. Bettinger Microfluidic thermally activated materials for rapid control of macroscopic compliance. *Adv. Funct. Mater.* **24,** 4860-4866 (2014).
15. C. Linghu, et al. Universal SMP gripper with massive and selective capabilities for multiscaled, arbitrarily shaped objects. *Sci. Adv.* **6,** eaay5120 (2020).
16. D. Yuan, et al. Biomimetic water-responsive self-healing epoxy with tunable properties. *ACS Appl. Mater. Interfaces* **11,** 17853-17862 (2019).
17. X. Zhao, et al. Phase change mediated mechanically transformative dynamic gel for intelligent control of versatile devices. *Mater. Horiz.* (2021).

18. Q. Zhou, et al. A Tailorable Family of Elastomeric‐to‐Rigid, 3D Printable, Interbonding Polymer Networks. *Adv. Funct. Mater.* **30,** 2002374 (2020).
19. W. Hu, et al. New dielectric elastomers with variable moduli. *Adv. Funct. Mater.* **25,** 4827-4836 (2015).
20. T. Nonoyama, et al. Instant thermal switching from soft hydrogel to rigid plastics inspired by thermophile proteins. *Adv. Mater.* **32,** 1905878 (2020).
21. L. Montero de Espinosa, W. Meesorn, D. Moatsou&C. Weder Bioinspired polymer systems with stimuli-responsive mechanical properties. *Chem. Rev.* **117,** 12851-12892 (2017).
22. K. Liu, et al. Biomimetic Impact Protective Supramolecular Polymeric Materials Enabled by Quadruple H-Bonding. *J. Am. Chem. Soc.* **143**, 1162-1170 (2020).
23. C. Zhao, X. Gong, S. Wang, W. Jiang&S. Xuan Shear Stiffening Gels for Intelligent Anti-impact Applications. *Cell Rep. Phys. Sci.* 100266 (2020).
24. P. Testa, et al. Magnetically Addressable Shape‐Memory and Stiffening in a Composite Elastomer. *Adv. Mater.* **31,** 1900561 (2019).
25. E. Brown, et al. Universal robotic gripper based on the jamming of granular material. *Proc. Natl. Acad. Sci. U. S. A.* **107,** 18809-18814 (2010).
26. E. Krieg, M. M. Bastings, P. Besenius&B. Rybtchinski Supramolecular polymers in aqueous media. *Chem. Rev.* **116,** 2414-2477 (2016).
27. W. Shan, S. Diller, A. Tutcuoglu&C. Majidi Rigidity-tuning conductive elastomer. *Smart Mater. Struct.* **24,** 065001 (2015).
