# Supplementary material for: Modulus adaptive lubricating prototype inspired by instant muscle hardening mechanism of catfish skin
Source: Nat Commun. 2022 Jan 19;13:377. doi: 10.1038/s41467-022-28038-9 (PMC8770490; doi:10.1038/s41467-022-28038-9)
Supplement: Supplementary file 3 — Description of Additional Supplementary Files [file 41467_2022_28038_MOESM3_ESM.pdf]

Description of Additional Supplementary Files

File name: Supplementary Movie 1

Description: Instant switch from soft, transparent state to rigid, opaque state of MALH.

File name: Supplementary Movie 2

Description: Shooting experiment of MALH bullets.

File name: Supplementary Movie 3

Description: Responsive lubrication behaviour of MASLD tested by artificial arms.

File name: Supplementary Movie 4

Description: Controllable movement of a mobile device on MALSD.

File name: Supplementary Movie 5

Description: The dynamic moving action of a submarine with MALSD caught by artificial arms.
